# Chronic histiocytic intervillositis: A breakdown in immune tolerance comparable to allograft rejection?

**DOI:** 10.1111/aji.13373

**Published:** 2020-11-24

**Authors:** Chloe A. Brady, Charlotte Williams, Megan C. Sharps, Amena Shelleh, Gauri Batra, Alexander E. P. Heazell, Ian P. Crocker

**Affiliations:** ^1^ Tommy's Maternal and Fetal Health Research Centre St. Mary’s Hospital The University of Manchester Manchester UK; ^2^ University of Exeter Exeter UK; ^3^ St Mary’s Hospital Manchester University NHS Foundation Trust Manchester UK; ^4^ Paediatric Histopathology Central Manchester University Hospitals NHS Foundation Trust Manchester UK

**Keywords:** graft rejection, HLA, macrophages, miscarriage, placenta, pregnancy, stillbirth

## Abstract

Chronic histiocytic intervillositis (CHI) is a pregnancy disorder characterized by infiltration of maternal macrophages into the intervillous space of the human placenta, often with accompanying perivillous fibrin deposition. CHI is associated strongly with foetal growth restriction and increased risk of miscarriage and stillbirth. Although rare, affecting 6 in every 10 000 pregnancies beyond 12 weeks’ gestation, the rate of recurrence is high at 25%–100%. To date, diagnosis of CHI can only be made post‐delivery upon examination of the placenta due to a lack of diagnostic biomarkers, and criteria vary across publications. No treatment options have shown proven efficacy, and CHI remains a serious obstetric conundrum. Although its underlying aetiology is unclear, due to the presence of maternal macrophages and the reported increased incidence in women with autoimmune disease, CHI is hypothesized to be an inappropriate immune response to the semi‐allogeneic foetus. Given this lack of understanding, treatment approaches remain experimental with limited rationale. However, there is recent evidence that immunosuppression and antithrombotic therapies may be effective in preventing recurrence of associated adverse pregnancy outcomes. With similarities noted between the pathological features of CHI and acute rejection of solid organ transplants, further investigation of this hypothesis may provide a basis for tackling CHI and other immune‐related placental conditions. This review will explore parallels between CHI and allograft rejection and identify areas requiring further confirmation and exploitation of this comparison.

## 1 INTRODUCTION

From implantation of the embryo through to birth, successful pregnancy relies upon the promotion of a tolerogenic uterine environment to allow the foetus to thrive.^1^ Simultaneously, and of equal importance, the maternal inflammatory response must be conserved to protect the mother and foetus against pathogens.^2^ This “paradox” of pregnancy, first described by Sir Peter Medawar in the 1950s,^1^ has prompted investigation into the immunology of pregnancy, particularly where it may be dysfunctional.

**FIGURE 1:**
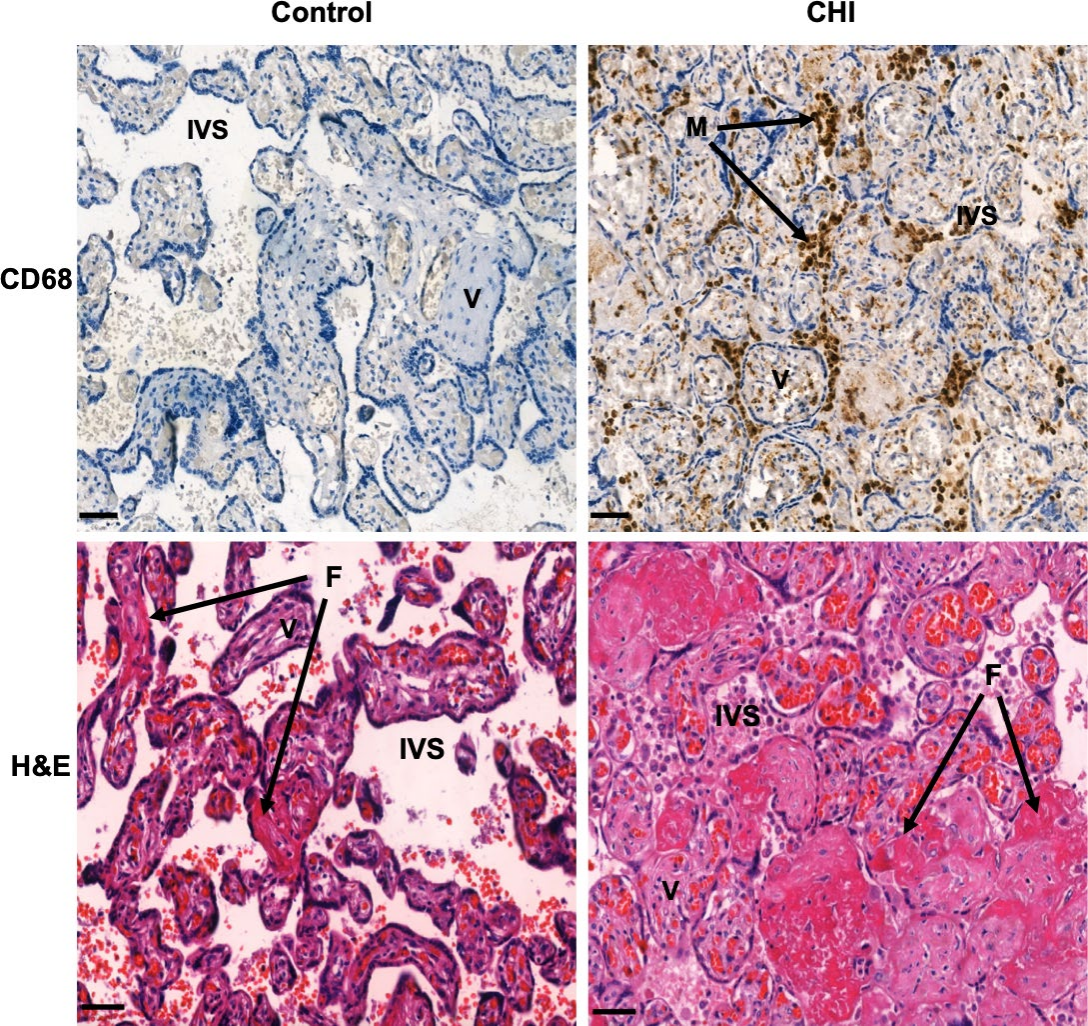
Histological characteristics of a placenta affected by chronic histiocytic intervillositis (CHI) compared with that of a healthy control pregnancy. Immunohistochemical staining demonstrates infiltration of maternal CD68^+^ macrophages (M) into the intervillous space (IVS) of the placenta in CHI, surrounding foetal villi (V). Fibrin (F) shown by haematoxylin and eosin (H&amp;E) staining as a shade of dark pink is present to a degree within the healthy term placenta, though is considerably increased in cases of CHI. Scale bars = 50μm

Chronic histiocytic intervillositis (CHI), also known as chronic intervillositis (CI), chronic intervillositis of unknown aetiology (CIUE) and massive chronic intervillositis (MCI),^3^ is an example of a rare placental inflammatory disease which can occur during any trimester of pregnancy, initially described by Labarrere and Mullen in 1987 as a placental lesion consisting of histiocyte (macrophage) infiltration within the intervillous space, fibrin deposition (Figure 1) and trophoblast necrosis.^4^ CHI is strongly associated with foetal growth restriction (FGR),^5^, ^6^ miscarriage and stillbirth.^7^, ^8^ A systematic review by Bos et al^3^ found the rate of live birth in cases of CHI to be 54.9%, with only 32.4% of pregnancies reaching term, possibly related to FGR and consequent intervention. The same review also found a miscarriage rate of 24%, with half occurring between 12 and 22 weeks’ gestation. FGR is reported to affect 42%–61.5% of pregnancies diagnosed with CHI.^5^, ^6^ Importantly, CHI has a 25%–100% chance of recurrence in subsequent pregnancies,^3^, ^6^, ^9^ with more severe or diffuse CHI associated with poorer outcomes.^10^ When infiltration of mononuclear cells and fibrin deposition have been graded in CHI cases,^11^ placentas with the lowest score corresponded to live births and those with highest grade have a 73% chance of *in utero* loss. A 2013 retrospective study from the Netherlands also correlated increased CHI severity with a shorter pregnancy duration and increased risk of miscarriage, stillbirth and neonatal death.^12^ A report also suggests that CHI may mimic features of osteogenesis imperfecta, including bone fractures in the foetus, though this was unable to be confirmed genetically.^13^ Three pregnancies studied in this report resulted in FGR and a small placenta, suggesting that foetal development may have been limited by uteroplacental insufficiency. CHI reportedly affects 6 in 10 000 s and third trimester placentas sent for histopathological examination^14^ and has been identified in 4.4% of first trimester miscarriages with normal karyotype.^15^ The suggested incidence of CHI in pregnancies with normal outcome is 0.2%–0.4%.^16^, ^17^

The exact mechanism by which CHI causes adverse outcomes is unknown. However, Marchaudon et al^8^ found that CHI‐affected pregnancies, complicated by spontaneous early miscarriage and FGR, were associated with more intense fibrin deposition within the placenta. In another comparison to healthy pregnancies, pregnancies with CHI demonstrated failure in physiological transformation of spiral arteries and a significantly higher presence of atherosclerotic‐like lesions,^18^ suggesting that nutrient and gas exchange across the placenta may be affected. Another study speculated that accumulation of cells within the intervillous space increases the oxygen diffusion distance between maternal erythrocytes and foetal villi,^19^ a source of reduced placental efficiency and dysfunction.

The pathophysiology of CHI has been sparsely described in the literature and remains poorly understood, perhaps understandable given its relative rarity and description since the early 2000s. Investigations of the underlying mechanisms are mostly based on retrospective case series, allowing limited interpretation and extrapolation. Clinical and histological observations from CHI patients indicate a disorder of immunological aetiology, though this is yet to be verified conclusively. This review appraises current literature regarding the pathophysiology, diagnosis and management of CHI. Furthermore, limitations in current evidence are considered and suggestions made for future research and its clinical management.

## 2 IMMUNE TOLERANCE IN HUMAN PREGNANCY

Historically, the ability of the mother to tolerate the semi‐allogenic foetus has led to the assumption that pregnancy is a state of immunosuppression.^20^ However, more recent research has shed light on pregnancy as a unique state of tolerance, requiring a careful balance of foetal evasion of the maternal immune system with appropriate and proportionate modulation of maternal immune cell function.^21^ Outside of pregnancy, non‐self‐antigens, such as those on the surface of pathogens or a transplanted organ, result in an inflammatory response.^22^ However, during pregnancy, immune tolerance results in the limitation of this response, allowing accommodation of the genetically different foetus across gestation.^21^

In brief, immune tolerance in pregnancy involves the expansion of immune modulating, anti‐inflammatory cells and cell functions. Amongst these are regulatory T cells (Tregs), which play an essential immunosuppressive role via secretion of anti‐inflammatory cytokines IL‐10 and TGF‐β, and limitation of T‐cell responses towards foetal antigens.^21^, ^23^, ^24^ Interestingly, Tregs demonstrate specificity towards foetal antigens and exhibit accelerated proliferation during future pregnancies.^25^ Following fertilization and embryo implantation, uterine natural killer cells (uNKs) form the dominant component of the immune milieu, though their cytotoxic effects towards trophoblast cells are attenuated via decidual macrophages.^26^ After uNKs, macrophages are the second most abundant decidual leucocyte and persist throughout pregnancy, with the majority polarized towards an M2 anti‐inflammatory phenotype.^27^ In rodent models, dendritic cells (DCs) are entrapped within the uterus preventing antigen presentation and may be responsible for the induction of Tregs from naïve T cells.^23^, ^28^ Stromal cells of the decidua may also contribute towards tolerance via silencing expression of genes related to chemokine production, as shown in mice.^29^

In addition to maternal adaptations, the trophoblast has evolved to evade immune detection, through limited expression of low‐immunogenic non‐classical self‐antigens (human leucocyte antigens, HLAs) and the immunomodulatory properties of expressed HLA‐G.^30^ Together, maternal immune cell adaptation and placental HLA expression demonstrate multiple protective mechanisms against both paternal antigen sensitization and maternal anti‐HLA antibody production, both known determinants of poor outcomes, including preterm delivery and chronic chorioamnionitis.^31^, ^32^ Control and appropriate timing of inflammation are crucial for all stages of healthy pregnancy, facilitating implantation, pregnancy maintenance and finally parturition.^33^

## 3 INFLAMMATION IN IMPLANTATION AND PARTURITION

Normally, physiological inflammation within the local uterine environment occurs at specific stages in pregnancy and is required for adequate placental development, as well as the initiation of parturition. Healthy pregnancy is therefore a balance of inflammatory and tolerogenic processes, in which a breakdown in either may have pathogenic consequences, including recurrent pregnancy loss (RPL), foetal immune activation in villitis of unknown aetiology (VUE) and CHI.

Endometrial studies have noted that implantation and placental development are associated with a strong pro‐inflammatory Th1 type response, characterized by increased pro‐inflammatory cytokines, IL‐6, IL‐8 and TNFα.^34^, ^35^ The production of these cytokines by endometrial cells is responsible for the recruitment of uNKs, DCs and macrophages to the decidua.^34^ An established inflammatory gradient has also been hypothesized for the production of increased adhesion molecules by endometrial epithelial cells in guiding the blastocyst to the implantation site and facilitating trophectoderm attachment.^34^

Following this inflammatory phase, recruited decidual leucocytes reportedly adopt a more immunomodulatory phenotype. uNKs are responsible for establishing instability within maternal vessels as a prerequisite to uterine vascular remodelling, promoting trophoblast invasion of the endometrium, via IFN‐γ release and production of angiogenic factors to enhance maternal blood flow.^36^, ^37^, ^38^ Like uNKs, DCs and macrophages begin to secrete angiogenic factors in addition to anti‐inflammatory cytokines (IL‐4, IL‐10 and IL‐13), and are responsible for the subsequent shift to an anti‐inflammatory Th2 profile, which predominates the remainder of pregnancy until parturition.^39^ Macrophages also play an important role in phagocytosis of cellular debris, reducing contact between foetal antigens and the maternal immune system during vascular remodelling, and continued trophoblast release from the placenta through cell turnover and vesicle production.^40^

Though the initiation of parturition is poorly understood, an inflammatory component has been consistently identified, alongside the roles of myometrial stretch, hormones and prostaglandins.^41^ An increase in circulating IL‐1, IL‐6 and TNF‐α has been identified in both spontaneous term labour and preterm birth, and is thought to regulate prostaglandin release and activation via the cyclooxygenase‐2 (COX‐2) pathway.^42^ The resulting cervical ripening, uterine contractions and placental detachment are all necessary components for normal birth.^42^ The extent and timing of this cytokine cascade inevitably requires precise control, as excess inflammation (ie generated by autoimmune disease) may instigate early‐onset delivery as epitomized in preterm labour.^33^, ^43^, ^44^

## 4 INFLAMMATION AND RECURRENT MISCARRIAGE

Recurrent miscarriage (RM), defined as three or more consecutive miscarriages, is a diverse condition in which more than half of couples have no identifiable cause.^45^ In such cases, immune dysfunction has been investigated as a possible explanation; low Treg levels, anti‐HLA antibodies and NK cell levels have also been postulated as a causative factor,^46^, ^47^ with several groups reporting increased NK cell populations in pre‐pregnancy endometrium of women with RM.^48^, ^49^, ^50^ Nevertheless, studies have been contradictory.^51^, ^52^ Immunosuppression using prednisolone treatment has been trialled in RM and achieved depletion in uNKs,^53^ but here again clinical efficacy is debated, especially given the recognized importance of uNK cells in the development of tolerance and healthy placentation.^54^

## 5 INFLAMMATION AND VILLITIS OF UNKNOWN AETIOLOGY

VUE is a placental inflammatory condition associated with FGR, stillbirth and possible neurological impairment.^55^, ^56^, ^57^, ^58^ Placentas with VUE exhibit infiltration of maternal CD8 + T cells into the chorionic villous tree and small number of maternal CD68 + macrophages, in addition to the activation of resistant placental villous macrophages, Hofbauer cells.^59^ Deposition of the complement protein C4d, a marker of innate immune activation, is also evident, as well as a Th1 pro‐inflammatory cytokine profile and upregulation of genes and chemokines associated with tissue rejection.^60^, ^61^ The primary cause of inflammation in VUE is unclear; however, it is known that cell death within the placenta induces release of damage‐associated molecular patterns (DAMPs) which alter the chemokine profile and result in recruitment of maternal immune cells.^33^ It may therefore be possible that VUE is a sterile inflammatory response to placental damage of unknown origin, occurring most commonly in the third trimester when tolerance begins to decline.^62^

## 6 INFLAMMATION AND CHRONIC HISTIOCYTIC INTERVILLOSITIS

CHI is an inflammatory lesion of mononuclear cell accumulation within the placenta.^4^ The predominant theory is it is a disorder of excessive maternal inflammation directed towards the placenta,^11^, ^63^ possibly occurring in early pregnancy, but exacerbated in the third trimester, where it is more readily identified. This theory is supported by the presence of fibrin deposition, complement activation and B‐ and T‐cell responses directed towards paternal antigens in CHI.^12^, ^63^ CHI has also been previously linked to foetal and neonatal alloimmune thrombocytopenia (FNAIT), a maternal immune response mounted against foetal platelet antigens.^64^ Other studies have also noted increased incidence in women with pre‐existing autoimmune disease.^65^, ^66^

## 7 PATHOPHYSIOLOGY OF CHI

**FIGURE 2:**
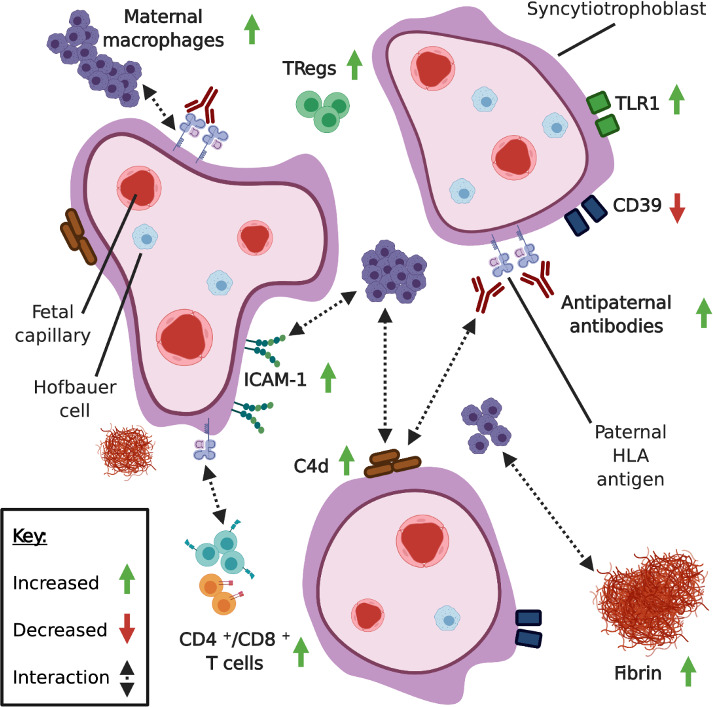
Suggested pathophysiology of chronic histiocytic intervillositis (CHI) of the placenta. Maternal monocytes, predominantly CD68 + M2‐like macrophages, infiltrate the intervillous space of the placenta, in association with increased fibrin deposition.^67^ CD4+ and CD8 + T cells are also increased within maternal blood surrounding the foetal villi, though they constitute a much smaller proportion of the infiltrate.^11^ In addition to T cells directed towards paternal antigens, anti‐HLA antibodies have been identified in women affected by CHI and are hypothesized to interact with antigens expressed on the placenta.^12^ In some cases of CHI, deposition of the complement protein C4d, usually associated with an antibody‐mediated immune response and macrophage recruitment, is increased along the surface of the syncytiotrophoblast.^63^ Increased intercellular adhesion molecule‐1 (ICAM‐1) expression by syncytiotrophoblast is also hypothesized as a contributory factor in macrophage recruitment.^75^ Expression of CD39, an ectonucleotidase responsible for hydrolysis of damage‐associated molecular patterns (DAMPs), is decreased in CHI in areas of dense cellular infiltration, suggesting it may also be involved in the maternal inflammatory response.^77^ The mechanism of fibrin deposition in CHI is unclear, though it is often present as a non‐specific response to placental damage.^111^ Macrophages in CHI have been found to express complement receptor CR4, which outside of CHI is capable of mediating monocyte adhesion to fibrinogen, though remains unexplored in this context.^67^ Certain cases of CHI demonstrate increased toll‐like receptor 1 (TLR1) expression, suggesting a possible bacterial component, though this is not always evident.^81^ Figure created with BioRender.com

Proposed pathophysiological pathways in CHI are summarized in Figure 2. The infiltrate consists of CD45+ and CD68 + monocytes and a small proportion of CD4+ and CD8 + T cells (Figure 2).^19^ CD68 + macrophages form the predominant component of the cellular infiltrate in CHI, though despite their abundance it is largely undetermined what effect they exert on the placenta and how they contribute towards adverse pregnancy outcomes.^19^

Characterization of the expression profile of maternal intervillous macrophages in CHI reveals an M2‐like anti‐inflammatory polarization state indicated by the expression of CD163.^67^ This finding is consistent with previous data, suggesting that macrophages in CHI are “resting,” without destructive infiltration into villous tissues.^19^ In the same expression analysis, CHI macrophages showed an overexpression of CD11c and CD18, which together form the complement receptor CR4.^67^ Though CR4’s role in CHI is yet to be investigated, more general studies on its function suggest it mediates monocyte adhesion to fibrinogen, the soluble component in blood which is converted to insoluble fibrin during clotting.^68^, ^69^ As increased fibrin deposition is widely described as a histopathologic feature of CHI,^4^, ^8^, ^70^ it is possible that its presence has an effect on monocyte accumulation and persistence in the intervillous space, but this proposal remains unexplored.

Reus et al^12^ detected a higher cytotoxic T lymphocyte precursor frequency (CTLpf) in women with CHI compared to controls, as well as the presence of partner‐directed anti‐HLA antibodies. In RM and preterm birth, the presence of anti‐HLA antibodies is associated with the reduced chance of a livebirth,^31^, ^47^ but in CHI it is not yet known whether these antibodies have the ability to bind to the placenta and, if so, what effect they have on its structure and/or function. The generation of partner‐directed anti‐HLA antibodies, albeit to a lower degree, is also a normal physiological response to healthy pregnancy following exposure to paternal antigens during conception and birth.^71^ Therefore, it remains to be confirmed whether these antibodies have clinical relevance in the development of CHI or are just a feature of the normal immune response in pregnancy.

Further evidence of a possible antibody‐mediated component to CHI is suggested by the presence of complement cascade split product C4d along the apical surface of the syncytiotrophoblast.^63^ C4d deposition is often used as a marker of activation of humoral immunity as it is produced following activation of the complement cascade classical pathway by both IgG and IgM.^72^ Complement activation via anti‐HLA antibody binding in other biological contexts, including in vitro models of solid organ rejection, appears to be responsible for increased adhesion of monocytes,^73^ and other products of the cascade influence both macrophage and T‐cell activity.^74^ Involvement of complement in CHI may represent a possible mechanistic link between antibodies in maternal serum and infiltration of macrophages and T cells into the placenta, though this remains speculative.

There are few studies of placental protein expression in CHI, but upregulation of intercellular adhesion molecule‐1 (ICAM‐1) on the syncytiotrophoblast has been noted.^75^ ICAM‐1 is responsible for the migration of leucocytes via the leucocyte function‐associated antigen‐1 (LFA‐1) and increases monocyte adhesion to the syncytial surface.^76^ In vitro, syncytiotrophoblast upregulate ICAM‐1 in response to the cytokines IL‐1α, IFN‐γ and TNF‐α, which are naturally produced during inflammation by macrophages and T cells.^76^ In CHI, the observed increase in ICAM‐1 may be either a causative factor, contributing to maternal immune cell recruitment into the intervillous space, or a consequence of placental damage.

In addition to ICAM‐1, a second protein implicated in immune cell recruitment in CHI is CD39, an ectonucleotidase normally expressed by trophoblast.^77^ CD39 hydrolyses ATP (adenosine triphosphate) which is released from damaged or apoptotic cells and acts as a DAMP.^78^ Therefore, ATP’s hydrolysis by CD39 acts as an immunosuppressive mechanism in healthy tissue.^77^ Sato et al^77^ found that CD39 expression was significantly decreased in 22 cases of CHI compared with controls. Furthermore, placentas from CHI pregnancies with poor outcomes (FGR and foetal death) demonstrated a greater decrease in CD39 expression and a significantly greater level of CD68 + cell infiltration compared with those resulting in livebirth. Regions of dense intervillous CD68 + macrophage infiltration also corresponded with areas of trophoblast which displayed reduced CD39. Though the mechanism of CD39 downregulation was not investigated in this study, the findings indicate that it may be a contributory factor in CHI pathogenesis and certainly warrants further investigation.

Another immunomodulatory factor implicated in CHI is CD200 (cluster of differentiation 200), a cell surface protein with immunosuppressive function responsible for the promotion of anti‐inflammatory M2 macrophages and Treg differentiation, as well as the inhibition of cytotoxic NK cell responses.^79^, ^80^ Preliminary data from a single case of CHI demonstrated reduced CD200 expression on syncytiotrophoblast compared with healthy term placenta.^79^ This may suggest a mechanism for inadequate tolerance in CHI as theoretically, a lack of CD200 should result in fewer Tregs. However, a study by Capuani et al^11^ revealed that the condition was associated with increased Treg populations in the decidua and intervillous space. The Treg number was positively correlated with CHI severity, and although their role is unclear, it is possible that their expansion represents an effort to resolve inflammation and breakdown in tolerance at the maternal‐foetal interface.^11^

As already described, the specific immunological trigger in CHI is unidentified, though a bacterial component has been speculated. Toll‐like receptor‐1 (TLR1) expressed by monocytes is involved in recognition of bacterial infections and lipopolysaccharide (LPS)‐induced inflammation, and in a study by Hussein et al^81^ was upregulated in certain cases of CHI. In diagnosis of placental disease, cases are initially screened in order to rule out common infectious agents, for example malaria, which exhibits a similar monocytic infiltrate^82^; however, the possibility of undetected bacteraemia has still been proffered.^81^ Not all cases of CHI in this study exhibited increased TLR1 expression, and it is unlikely that the recurrent nature of CHI can be explained by a bacterial agent, so it is important that other main pathways of non‐infectious pathogenesis are considered.

## 8 CHI AND OTHER INFLAMMATORY PLACENTAL LESIONS

CHI can be associated with other inflammatory lesions of the placenta including VUE and massive perivillous fibrin deposition/maternal floor infarction (MPFD/MFI). Similarities and differences between these lesions are summarized in Table 1. Importantly, these lesions are distinguished from those with infectious cause, such as malaria and chorioamnionitis which result in placental infiltration of maternal macrophages and neutrophils, respectively.^83^, ^84^

**Table 1:** Comparison of non‐infectious inflammatory placental lesions

	CHI	VUE	MPFD/MFI
Definition	Non‐infectious infiltration of the intervillous space by maternal mononuclear cells, with or without associated fibrin deposition^112^	Non‐infectious mononuclear cell infiltrate into the villous stroma, with destruction/necrosis of villous parenchyma^113^	Extensive deposition of fibrin within the intervillous space, or within and around the basal plate^111^
Incidence	0.01% of placentas^3^	5%–15% of placentas^62^	0.028%–0.4% of pregnancies^16^, ^114^, ^115^
Rate of recurrence	25%–100%^3^, ^6^, ^9^	10%–15%^62^	12%–78%^114^
Associated adverse pregnancy outcomes	FGR, preterm birth, foetal death^3^	FGR,^116^ stillbirth and neurological impairment^10^, ^55^, ^57^	FGR,^114^ preterm birth,^114^ foetal death,^117^ foetal malformations and neurological impairment^118^, ^119^
Histological features	Trophoblast necrosis^4^ Infiltrate composed mainly of CD68 + M2‐like macrophages^19^ Fibrin deposition^3^	Infiltrate composed mainly of maternal CD8 + T cells^59^ Activation of foetal macrophages—Hofbauer cells^59^	Fibrin deposition resulting in engulfment and atrophy of chorionic villi^111^
Evidence for immunological aetiology	Association with autoimmune disease/autoantibodies^65^, ^66^ C4d deposition^63^ Increased maternal CTLpf^12^ Increased anti‐HLA antibodies^12^ Syncytiotrophoblast ICAM‐1 upregulation ^75^ Placental CD39 downregulation^77^	Th1 pro‐inflammatory cytokine profile^120^ C4d deposition^60^ Upregulation of graft rejection‐associated genes and chemokines^61^ Upregulation of class I and class II HLA in inflamed villi^61^	Association with autoimmune disease/autoantibodies^111^ C4d deposition^121^ Increased anti‐HLA antibodies^88^, ^121^ Increased inflammatory chemokine expression^88^

Note

Chronic histiocytic intervillositis (CHI), villitis of unknown aetiology (VUE) and massive perivillous fibrin deposition (MPFD/MFI) have reported overlaps in pathology, with evidence of suggested immune involvement and rejection.

Abbreviations: CD, cluster of differentiation; CTLpf, cytotoxic T lymphocyte precursor frequency; FGR, foetal growth restriction; HLA, human leucocyte antigen; ICAM‐1, intercellular adhesion molecule‐1.

In some instances of CHI, VUE is also evident, with combined lesions in 25%–47% of cases.^10^, ^75^, ^85^ Due to their concurrence, it has been suggested by Nowak et al^10^ that the two disorders are on the same spectrum of disease, where CHI is the more extreme variant, causing higher morbidity and earlier presentation.^7^, ^10^ In support of this hypothesis, FGR is reported to be more frequent in CHI than VUE and is associated with a five times higher risk of stillbirth (29.2% vs. 6.4%).^10^ VUE also recurs in subsequent pregnancies, with an increasing risk of FGR and stillbirth.^58^ There are important differences between CHI and VUE which cast doubt on the hypothesis that they are related disorders. Firstly, VUE is more frequent than CHI, affecting up to 15% of all placentas.^62^ Inflammation in CHI is limited to the maternal placental intervillous compartment rather than infiltration of the chorionic villous tree and stroma, typical of VUE. There is also a lack of Hofbauer cell activation.^19^ Although both disorders similarly exhibit a mononuclear inflammatory infiltrate, CHI is mainly composed of maternal macrophages with a smaller proportion of CD8 + T cells.^10^ Labarrere et al^85^ described both lesions as containing a higher proportion of CD8 + T cells compared with CD4 + T cells; however, another study by Capuani et al^11^ found the ratio to be almost equal. Cytokine profiles in CHI and VUE have been compared by Freitag et al^19^ wherein CHI demonstrated lower expression of pro‐inflammatory cytokines, including CCL2 compared with VUE. This finding has been hypothesized as an explanation for the lack of destructive action of mononuclear cells in CHI, though the study was limited in size (N = 5), and thus requires further investigation and confirmation.^19^

Case reports have also described overlap of CHI with MPFD/MFI.^86^, ^87^ Like CHI, MPFD and MFI have been hypothesized as a possible manifestation of a maternal rejection‐type response towards the placenta, with increased anti‐HLA antibodies towards foetal antigens, C4d deposition and chemokine upregulation in certain cases.^88^ Other similarities noted between MPFD and CHI include risk of recurrence and an association with autoimmune disease or autoantibodies.^89^ The exact cause of MPFD is unknown, though studies have suggested a link to abnormalities in blood coagulation,^90^ a theory which has also been proposed in CHI with regard to the observed increase in fibrin deposition.^89^ Further work is required to establish whether these diseases are truly related, or separate entities with differing pathophysiology.

## 9 DIAGNOSIS OF CHI

Both VUE and CHI are asymptomatic for the mother and can occur across different maternal ages and in women with a range of obstetric histories.^3^, ^19^ Currently, they can only be diagnosed upon histopathological examination of the placenta after delivery. For CHI, there is no standardized classification by which it is graded; therefore, scoring systems validated in several different patient populations are required.^3^, ^10^ Pathologists in two studies graded CHI according to level of severity observed: absent, focal (<10% of the slide), moderate (10%–50%) and severe or massive (>50%).^9^, ^12^ Reus et al^12^ suggested this as a reliable technique for CHI detection, despite the inter‐observer agreement being Κ = 0.54 (moderate). A systematic review by Bos et al^3^ found that the only agreed criterion, amongst 18 studies on CHI, was the presence of intervillous infiltrate, with only 61% ruling out infectious cases, and many excluding cases with villitis. This suggests that the reported incidence of CHI may fluctuate depending on varied inclusion and exclusion criteria between centres. Based on these findings, Bos et al^3^ proposed alternative standardized criteria for diagnosing CHI:

1. An infiltrate present in the intervillous space (most important requirement).
2. Approximately 80% of the mononuclear cells in the intervillous space being CD68^+^.
3. The occupied infiltrate being 5% or more of the total intervillous space.
4. Exclusion of cases with clinical or histopathological signs of infection.

A standardized approach to the diagnosis of CHI is needed to define critical values to inform future patient care,^91^ to reduce heterogeneity between studies, and allow more reliable comparisons in the underlying pathophysiology of CHI and its treatment. Besides the lack of robust diagnostic criteria, there is also a need to identify possible prenatal (or even preconception) biomarkers of CHI. Currently, reliance on histopathology for diagnosis means that intervention can only be initiated in subsequent pregnancies of women with poor outcomes attributed to CHI in a previous pregnancy. In particular, it is reported that serum alkaline phosphatase (ALP) is increased in some cases of CHI^5^, ^8^, ^92^ as well as FGR and preterm delivery.^93^, ^94^ though whether these changes have any predictive value is unknown.^95^ An observational study which investigated markers in CHI, including biochemical (eg pregnancy‐associated plasma protein‐A (PAPP‐A)) and radiological (placental dimension and uterine artery Doppler) features, found no assessment was consistently linked to diagnosis following delivery.^96^ Nonetheless, such markers may help to elucidate pathophysiology and inform individual patient care, justifying closer prenatal monitoring.

## 10 TREATMENT OF CHI

Due to CHI’s high recurrence, the use of pharmacological treatments including aspirin, low molecular weight heparin (LMWH) and/or steroids in future pregnancies has been proposed.^6^ Contro et al^6^ systematically reviewed the literature prior to 2010, which revealed 13 cases of CHI where immunosuppressive and/or thrombo‐prophylactic intervention were trialled. There was no significant difference between livebirth rates in the treatment group compared with untreated CHI cases. However, the treatment regimens were inconsistent and small study sizes limited detailed analysis of therapeutic combinations. A case report by Ozawa et al^5^ described prophylactic treatments of a woman with previous CHI‐related recurrent miscarriage, severe FGR and a stillbirth at 27 weeks’ gestation. In this case, CHI occurred in her next pregnancy despite aspirin monotherapy, with diffuse presentation and moderate fibrin deposition.^5^ For this woman, a combination of aspirin and heparin was given during her subsequent pregnancy; FGR occurred but the patient delivered a live infant at 33 weeks’ gestation, with the placenta again showing diffuse macrophage infiltration and moderate fibrin deposition. Using a combination of prednisolone and low‐dose aspirin in another subsequent pregnancy, no FGR was noted and a live infant was delivered. The placenta showed focal CHI and mild fibrin deposition only. Although the pregnancy that used prednisolone and low‐dose aspirin in combination held the most favourable outcome and least severe CHI, conclusions should not be drawn from this single case report.

A prospective study by Mekinian et al^66^ investigated potential treatments for the prevention of CHI in 24 women with a previous diagnosis and included a variety of treatment regimens: aspirin or LMWH monotherapy, aspirin/prednisone dual therapy, aspirin/LMWH/prednisone polytherapy and aspirin/LMWH/prednisone/hydroxychloroquine polytherapy. No particular treatment regime was found to improve pregnancy outcomes, though again this study was limited by a small sample number in each treatment group and possible bias, given that polytherapy was used more frequently in women with more severe obstetric history.

Given the rarity of CHI, it is difficult to explore interventions and ethical issues surrounding randomized placebo‐controlled studies in women who often have extremely poor obstetric histories. Evidence surrounding current therapies is limited, and no single treatment demonstrates clear beneficial effects.^5^ The justification for use of immunosuppressive therapies in CHI is based on increasing evidence that it is a disease of maternal anti‐foetal rejection, though the specific pathophysiology, as discussed already, is largely unknown. Comparing similarities between CHI and organ rejection could allow potential exploratory avenues and direct research into more targeted and effective treatments, once causative mechanisms are identified.

## 11 SIMILARITIES BETWEEN ALLOGRAFT REJECTION AND CHI

As the foetus expresses paternal antigens, akin to a semi‐allograft and requiring maternal immune tolerance, the state of pregnancy has long been likened to recipient acceptance of a transplanted donor organ.^1^ In this comparison, the presence of anti‐paternal T cells and antibodies in CHI patients^12^, ^19^ draws parallels with the donor‐directed immune response in acute graft rejection, which unlike CHI has been better characterized, with attributed diagnostic criteria.^97^

**FIGURE 3:**
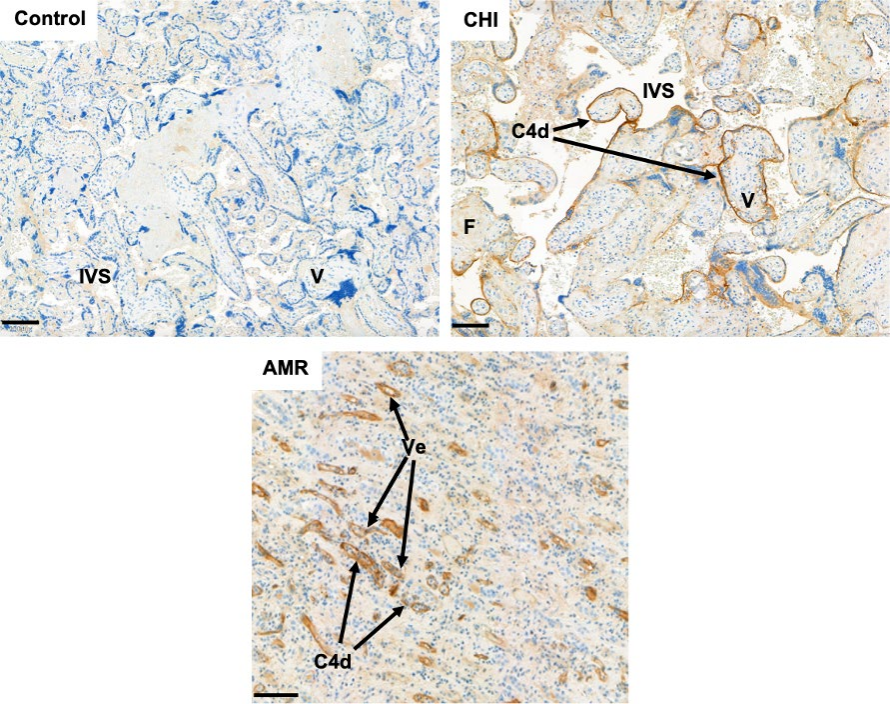
Immunohistochemical staining of complement cascade split product C4d in a healthy placenta and a case of chronic histiocytic intervillositis (CHI), compared with a biopsy of a kidney with confirmed antibody‐mediated rejection (AMR). In some cases of CHI, C4d is present along the apical membrane of the syncytiotrophoblast, similar to deposition within the vessels (Ve) of a rejected kidney allograft. Within placentas affected by CHI, terminal villi are often entrapped within deposits of fibrin (F). IVS = intervillous space, V = villi. Scale bars = 100μm

Histopathological features common to both allograft rejection and CHI include macrophage infiltration and deposition of fibrin and complement (Figure 3), ultimately resulting in dysfunction of the semi‐allogeneic organ,^98^ which in CHI may be comparable to the failure of the placenta to maintain foetal growth and/or survival. Graft rejection can be classified as antibody‐mediated rejection (AMR) or cellular rejection, though it is possible for both to coexist.^99^ In AMR, donor‐specific antibodies (DSAs) are either pre‐formed following sensitization events (eg pregnancy, blood transfusion or previous transplant)^100^ or produced de novo by B cells and plasma cells in response to HLA antigens on the vasculature of the allograft.^101^ AMR leads to graft loss via complement deposition, recruitment of NK cells, monocytes and macrophages which contribute towards fibrin formation, endothelial cell damage and eventually interruption of vascular function.^98^, ^102^ Anti‐HLA antibodies identified in CHI could be likened to these DSAs, especially as complement, monocytes and macrophages also appear to play a key role in its pathophysiology.^4^, ^63^ However, as the placenta is known to express only the least polymorphic of HLA molecules with low immunogenicity, it is unclear how anti‐HLA antibodies may be detrimental when not deemed so in normal healthy pregnancy.^71^ On the other hand, in VUE, Enninga et al noted increased placental expression of class I and class II HLA molecules along with upregulation of genes normally associated with graft rejection,^61^ though this was within foetal villi as opposed to the syncytiotrophoblast in contact with the maternal circulation, where CHI is always focused. Such studies into VUE have strengthened the comparison with allograft rejection, though other groups have argued the disorder simultaneously resembles graft‐vs‐host disease given the coexistence of a foetal inflammatory response.^55^, ^103^ Currently, evidence for VUE as a form of rejection is greater compared with CHI (perhaps due to differences in incidence), though this hypothesis still remains to be proven. It may therefore be worthwhile to undertake similar studies in CHI, including the aforementioned genetic analysis, to determine whether these inflammatory processes are also common to this disorder.

Cellular rejection in a transplanted organ is characterized by CD8 + T‐cell activation and the production of pro‐inflammatory cytokines resulting in cytotoxic effects.^104^ The action of T cells in cellular rejection can also be responsible for the recruitment of other effector cells, including macrophages, which further implement vascular injury.^105^ CD8 + T cells have been observed in CHI, though their role appears minor in comparison with that of the M2 macrophages which dominate the cellular infiltrate.^11^ However, compared with graft rejection, the roles of T cells and macrophages and their possible interaction in CHI remain poorly defined and require further study.

As some patients with CHI exhibit features consistent with either AMR or cellular rejection or both, it is possible that multiple pathological processes occur in the manifestation of CHI and may vary between individuals. This could explain the differing success of immunosuppressive therapies observed in CHI, and why particular therapies thought to target T‐ and B‐cell responses, such as hydroxychloroquine, are reportedly effective in certain cases, but not others.^106^ Similarities between rejection and CHI may suggest a potentially lucrative investigative avenue to frame future CHI research, though in comparison with VUE these have been explored to a much lesser extent. Considering the rarity of CHI, which limits current evidence base, the use of a model or framework on which to build future studies may expedite research and findings in the field.

## 12 FURTHER INVESTIGATIONS

Much is still unknown about CHI, and current evidence is significantly limited by small sample sizes. Although rare diseases tend to have weaker evidence, there are methods of overcoming these difficulties; encouragement to thoroughly investigate placental pathology in RM, FGR and stillbirth with subsequent multicentre studies would improve epidemiological knowledge and the robustness of studies performed. Moreover, prospective studies of unselective samples, determining the full spectrum of prognosis associated with CHI, as well as identifying a clear relationship between the intervillous infiltrate and pregnancy outcomes, would reduce the possibility of selection or reporting bias.

Important clinical questions remain, including which factors predict disease recurrence and the relationship between severity of histopathological findings and pregnancy outcomes, to assist in the assessment and management of patients with a history of CHI. Additionally, studies investigating the clinical and aetiological significance of CHI, VUE and MPFD in discrete and combined lesions are required, to ascertain whether these conditions are related or distinct entities.^10^ It may be valuable to investigate whether all pregnancies of mothers with a history of CHI require closer monitoring, or whether this is the case only if the previous‐defined disease was diffuse in the placenta or the pregnancy had a poor outcome.^10^

There is also an important need to identify risk factors for the development of CHI, in order to discriminate which women are likely to mount an inappropriate immune response to their foetus, prenatally or possibly even pre‐conceptionally. In order to do so, future studies are required to characterize the full range of clinical presentations of CHI, as well as potential prognostic biomarkers. Utilization of pre‐transplantation testing may prove useful here, as advances in the prediction and prevention of rejection means graft survival can be maintained for years using medication even in recipients with a high degree of HLA mismatch to their donors, and may therefore also be possible in CHI.^107^

Aside from a lack of prognostic biomarkers and a treatment regime with proven efficacy, the fundamental background knowledge on the causes of CHI remains limited. Its similarities shared with organ rejection suggest that this may be used as a model of immune tolerance breakdown to direct research into CHI pathophysiology and management. In this model, CHI‐affected placentas and maternal serum from such cases could be assessed in a similar fashion to other specialists in the investigation of organ rejection. For example, using the cross‐matching process to pinpoint causative antibodies in sera of CHI patients. Additionally, undertaking assessments of organ function, such as imaging and serum analysis currently exercised in transplantation,^108^, ^109^ may help find indicators of placental dysfunction of value in all areas of placental research. Applying an established evidence base to a rare condition could accelerate the investigative process and allow for earlier detection, not only in CHI but also other obstetric conditions with a suspected immune component. Further to this, a strong rationale for the use of screening, immunosuppressive therapies and increased monitoring in CHI, could be provided to prevent subsequent adverse outcomes.

## 13 CONCLUSION

CHI is a rare but serious and recurrent cause of RM, FGR and stillbirth, but evidence for its aetiology, presentation, diagnosis and management is weak. Current research points towards a maternal immunopathological response as the underlying cause.

In tackling this placental inflammation, some evidence of improved pregnancy outcomes is seen using immunosuppressive therapies and closer monitoring of foetal growth.^66^, ^110^ There is a need for predictive or diagnostic antenatal testing to identify CHI and its severe outcomes, but the rarity and limitations of the current evidence base restrict advancements in this area. A potential method to improve understanding of CHI is to model the disease on another process: one with a more robust evidence base, for example allograft rejection. To do so, more extensive study is required into the immunological mechanisms of CHI, on which knowledge is currently lacking. In defining any commonalities between CHI and rejection, such approaches could exploit the wealth of knowledge and technological advances already employed in transplant biology.

In future, collaborations between multiple centres that research and/or treat CHI is essential to expand sample sizes and identify mechanisms of pathogenesis. In doing so, the benefit of increased knowledge of CHI will likely extend to other obstetric disorders and reduce the high risk of recurrence and distress in affected women and their partners.

## Data Availability

Data sharing is not applicable to this article as no new data were created or analyzed in this study.
